# Personalized diversification of complementary recommendations with user preference in online grocery

**DOI:** 10.3389/fdata.2023.974072

**Published:** 2023-03-22

**Authors:** Luyi Ma, Nimesh Sinha, Jason H. D. Cho, Sushant Kumar, Kannan Achan

**Affiliations:** ^1^Walmart Global Tech, Sunnyvale, CA, United States; ^2^DoorDash, San Francisco, CA, United States

**Keywords:** diversification, re-ranking, recommender system (RS), complementary recommendation, personalization

## Abstract

Complementary recommendations play an important role in surfacing the relevant items to the customers. In the cross-selling scenario, some customers might present more exploratory shopping behaviors and prefer more diverse complements, while other customers show less exploratory (or more conventional) shopping behaviors and want to have a deep dive of less diverse types of complements. The existence of two distinct shopping behaviors reflects users' different shopping intents and requires complementary recommendations to be adaptable based on the user's shopping intent. Although many studies focus on improving the recommendations through post-processing techniques, such as user-item-level personalized ranking and diversification of recommendations, they fail to address such a requirement. First, many user-item-level personalization methods cannot explicitly model the preference of users in two types of shopping behaviors and their intent on the corresponding complementary recommendations. Second, most of the diversification methods increase the heterogeneity of the recommendations. However, users' intent on conventional complementary shopping requires more homogeneity of the recommendations, which is not explicitly modeled. The present study tries attempts to solve these problems by the personalized diversification strategies for complementary recommendations. To address the requirement of modeling heterogenized and homogenized complementary recommendations, we propose two diversification strategies, heterogenization and homogenization, to re-rank complementary recommendations based on the determinantal point process (DPP). We use transaction history to estimate users' intent on more exploratory or more conventional complementary shopping. With the estimated user intent scores and two diversification strategies, we propose an algorithm to personalize the diversification strategies dynamically. We demonstrate the effectiveness of our re-ranking algorithm on the publicly available Instacart dataset.

## 1. Introduction

Recommender system is an essential part of the e-commerce business. Recommending relevant items to customers makes the shopping experience more comfortable and time-saving. Online grocery platforms also have a wide variety of recommendation systems placed at various sections of their websites to improve customer journey. One of the important sections is related to complementary item recommendations for cross-selling. Given a query item, complementary recommendations show the query item's complements to customers who frequently co-purchase to fulfill a particular demand. For example, when a customer purchases a bag of hot dog, she/he might also want to purchase a bag of hot dog buns together. Showing hot dog bun for hot dog as a complementary item recommendation will improve the shopping experience.

**Figure 1:**
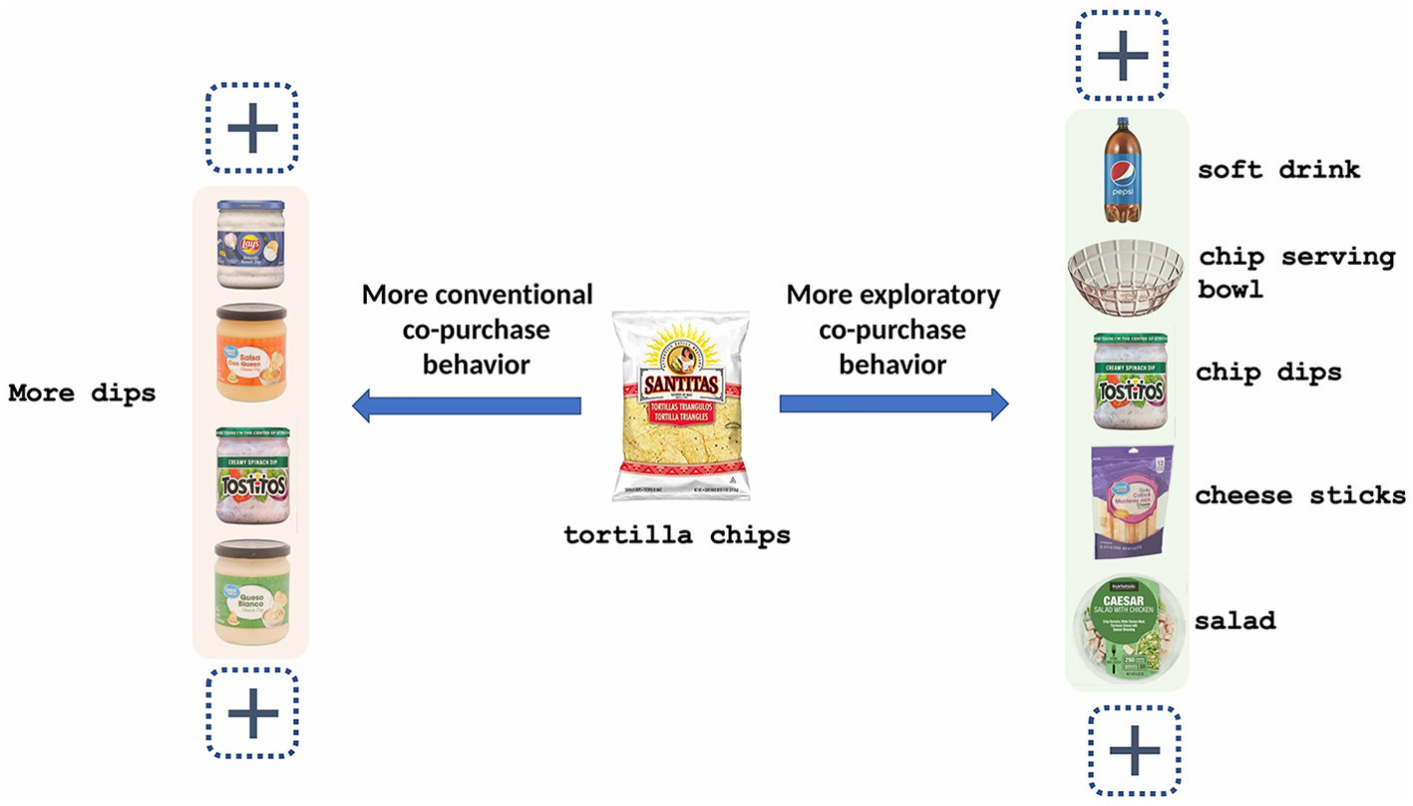
Exploratory co-purchase behaviors vs. Conventional co-purchase behaviors.

However, it is non-trivial to effectively recommend complementary items for a given query item when the users show different co-purchase behaviors, i.e., more exploratory co-purchase or more conventional co-purchase as shown in Figure 1. When a user prefers more exploratory co-purchases, she/he might also prefer to see more heterogeneous item recommendations complementary to the query item because of the intent on exploration. When a user prefers more conventional co-purchases, she/he might favor less diversified or even homogeneous item recommendations complementary to the query item because of the intent on classic combination with a deep comparison. In this case, the diversity of complementary item recommendations should be adaptable to the co-purchase pattern (exploratory vs. conventional) with personalization. Such an adaptation requires not only modeling the diversification of complementary item recommendations for more exploratory shopping intent, but also properly homogenizing the recommendation for the conventional shopping intent. Furthermore, we need to personalize the adaptation for users by their shopping intent.

All these problems become more challenging for online grocery because grocery items are deeply involved in our daily life under so many co-purchase scenarios, such that co-purchase patterns are more diverse and flexible compared with other online marketplaces. For example, in online grocery, tortilla chips have many food complements, such as salsa dip, guacamole dip, and soft drink and also non-food complements such as chip bowl, while in online electronics e-commerce, television (TV) might only have fixed complements related to television shopping.

**Figure 2:**
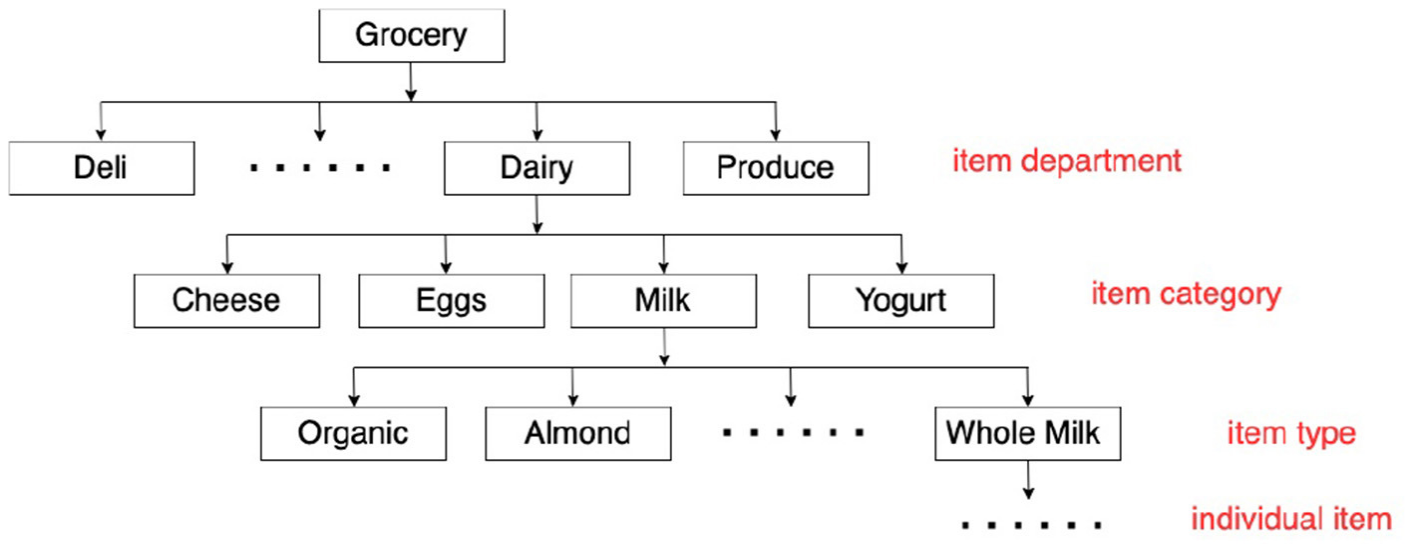
An example of grocery item taxonomy.

Diversity of item recommendations could be quantified by item attributes. One of the commonly used attributes is the hierarchical classification of an item in the taxonomy. Figure 2 presents an example of grocery item taxonomy, with *item department, item category, item type*, and *individual items*. While items from the same category (one level of item classification in the taxonomy) generally share a similar item functionality (e.g., items from the milk category), the department level classification summarizes the diversity of the customer shopping intent better because each department could represent an aspect of daily shopping. In the aforementioned example of tortilla chips, the customer needs to purchase items from multiple departments such as *Deli* and *Beverage*. In our case, we define the diversity of complementary items at the department level (i.e., only items from two different departments contribute to the increment in the recommendation diversity).

Many complementary item recommendation models mainly focused on learning the complementarity between items rather than the personalized adjustment of diversity of complementary item recommendations (McAuley et al., 2015; Barkan and Koenigstein, 2016; Wan et al., 2018; Wang et al., 2018; Zhang et al., 2018; Xu et al., 2019; Liu et al., 2020). Diversification of complementary item recommendations has been recently addressed in Hao et al. (2020) by considering the item type and categories. Unfortunately, it cannot distinguish the demand of users' shopping intent by surfacing more heterogeneous complementary items for exploratory shopping intent or more homogeneous complementary items for conventional shopping intent.

To address these challenges, we utilize the complementary item recommendations by the existing models and deploy the re-ranking strategy to balance out both the exploratory and the conventional complementary shopping intent. To illustrate the necessity and effectiveness of adjustable diversification of complementary item recommendations, we study two diversification strategies, **heterogenization** and **homogenization**. For heterogenization, we focus on diversified complementary item recommendations. We use the re-ranking strategy based on determinantal point process (DPP) to diversify our complementary item recommendations by the existing models. The more diversified complementary item recommendations can fit the exploratory shopping intent. For homogenization, we enforce the homogeneity of the complementary item recommendations by a re-ranking strategy based on a modified DPP. In this case, the modified DPP will encourage the homogeneity of the recommendations for conventional shopping intent.

To further address the personalized adjustment of diversification strategies, we estimate the user shopping intent (exploratory vs. conventional) by user shopping history. The estimated user shopping intent will guide the recommender system to select the proper diversification re-ranking (heterogenization vs. homogenization) for the complementary item recommendations and address the user shopping intent. We summarize our contributions as follows:

- We introduce the concept of exploratory and non-exploratory shopping demands from customer behavior to the modeling problem of complementary item recommendations, which has not been addressed before.
- We further address the requirement of personalizing the demand of exploratory and non-exploratory recommendations based on the diversity of recommendations and proposed a personalized ranking model for complementary item recommendations for the dynamic adjustment.
- We show the effectiveness of our proposed solution and conducted case studies on customer shopping intent on the publicly available dataset.

The rest of this article is structured as follows: we summarize the related articles in Section 2 and introduce the preliminaries of our model in Section 3. After that, we propose our model in Section 4. We provide the evaluation and result analysis in Section 5 and conclude our article in Section 6.

## 2. Related works

### 2.1. Complementary recommendations

Many studies have focused on the complementary item recommendations. Embedding-based methods, such as Barkan and Koenigstein (2016) and Wan et al. (2018), collaboratively learn the complementary item relationship from the co-purchase data. Another way of using the co-purchase data is to construct the co-purchase graph and apply graph neural networks on it (McAuley et al., 2015; Wang et al., 2018; Liu et al., 2020). They use the co-purchase records as labels for link predictions based on the distance between item embeddings. In addition to vector item embeddings, Gaussian embedding is also explored in Ma et al. (2021) to address the noise in the co-purchase data for better complementarity learning. In addition to the co-purchase data, many types of auxiliary data are incorporated into the modeling, such as the multimodal data of items (Zhang et al., 2018) and the shopping context (Xu et al., 2019). Diversified complementary recommendation is studied in Hao et al. (2020) by leveraging the product-type information to improve the diversity. However, it focuses on the diversified recall process rather than the ranking process as our article targets.

In our study, we leverage the **triple2vec** in Wan et al. (2018) to learn the complementary item embedding due to its effectiveness in learning the item vector embeddings from the co-purchase data.

### 2.2. Recommendation diversification

For a long time, not much importance was given to diversity in the recommendations, as it is challenging to achieve both high accuracy and diversity at the same time. This is called *accuracy diversity dilemma* (Liu et al., 2012). Novelty and diversity of items have been improved by penalizing accuracy (Díez et al., 2019). Diversity has also been captured in an entropy regularizer (Qin and Zhu, 2013). Post-processing methods for diversity have been proposed to improve the personalized recommendations generated by collaborative filtering (Adomavicius and Kwon, 2012). Determinantal point process (DPP) has been used for making personalized diversified recommendations and DPP models are probabilistic models with a lot of applications (Kulesza and Taskar, 2012). They have been incorporated with a tunable parameter allowing the users to smoothly control the level of diversity in recommendations and also, applied to large-scale scenarios with faster inference (Wilhelm et al., 2018). Deep reinforcement learning has utilized DPP to promote diversity to generate diverse, while relevant item recommendations. DPP kernel matrix is maintained for each user, which is constructed from two parts: a fixed similarity matrix capturing item-item similarity and the relevance of items dynamically learnt through an actor-critic reinforcement learning framework (Liu et al., 2019). However, they fail to give much attention to maintaining the delicate balance between the requirement of distinct diversity strategies for the exploratory and conventional shopping intents. Our proposed method focuses on the combined re-ranking strategy for exploratory and conventional user shopping intent on complementary recommendations.

## 3. Preliminaries

In this section, we first revisit the base model for complementary item recommendations, **triple2vec** (Wan et al., 2018), and for generating the item embedding used for diversification. We choose **triple2vec** as our baseline model for complementary item recommendation because we do not assume that transaction data (i.e., product IDs and user IDs) are the only available input due to its high accessibility for e-commerce systems and that there are no additional contexts such as click/view and user profiles (e.g., age and gender). Then, we introduce DPP for recommendation diversification and its basic setting.

### 3.1. Skip-gram-based item embedding and *triple2vec*

Skip-gram-based methods for item embedding leverage the item co-occurrence signal (e.g., co-purchase of items). Models for complementary item recommendations such as McAuley et al. (2015) and Barkan and Koenigstein (2016) exactly use the item co-occurrence signal to model item complementarity. **triple2vec** in Wan et al. (2018) introduced the cohesion of (*item, item*, and *user*) triplets that reflect the co-purchase of two items by the same user in the same basket. This technique improves the performance of complementary item recommendations and **triple2vec** achieves the state-of-the-art performance. As we focus on the post-processing of the recommendations, we decide to leverage the item representations learned by **triple2vec** to generate item pools for downstream applications.

In **triple2vec**, a triplet of (*q, r, u*), *q* ∈ *V, r* ∈ *V, u* ∈ *U*, represents the user-item and the item-item relationship, where *V* is the set of items and *U* is the set of users. Here, *q* and *r* are two items purchased by the user *u* in the same basket. Particularly, we refer *q* to the query item and *r* to the recommended item. The relationship between *q* and *r* can be viewed in the way that *r* is the recommended complementary item for the query item *q*. The cohesion of (*q, r, u*) in **triple2vec** is computed by Equation (1), where *f*_*q*_, *g*_*r*_ are two sets of representations for items (*q, r*) and *h*_*u*_ is the user embedding.


(1) $$\begin{array}{l} s_{q,r,u}=\overset{x}{\overset{︷}{f_{q}^{T}g_{r}}}+\underset{y}{\underset{︸}{f_{q}^{T}h_{u}+g_{r}^{T}h_{u}}} \end{array}$$

*x* and *y* in Equation (1) indicate the item-to-item complementarity and user-to-item compatibility, respectively. The loss function L in Equation (2) computes the likelihood of all possible triplets T and is optimized to learn representations of items and users.


(2) $$\begin{array}{l} L=\underset{q,r,u\in T}{\sum }\left(\log p\left(r|q,u\right)+\log p\left(q|r,u\right)+\log p\left(u|q,r\right)\right) \end{array}$$

Here, $p\left(r|q,u\right)=\frac{\exp\left(q,r,u\right)}{\underset{r^{\prime }}{\sum }\exp\left(q,r^{\prime },u\right)}$, $p\left(q|r,u\right)=\frac{\exp\left(q,r,u\right)}{\underset{q^{\prime }}{\sum }\exp\left(q^{\prime },r,u\right)}$, and $p\left(u|q,r\right)=\frac{\exp\left(q,r,u\right)}{\underset{u^{\prime }}{\sum }\exp\left(q,r,u^{\prime }\right)}$.

We leverage **triple2vec** to learn item representations and generate item pools of complementary recommendations for downstream processes. To recall the item pool of complementary recommendations, we consider the inner product score $f_{q}^{T}g_{r}$ for two items *q, r*. For each query item *q*, we select a pool of items *R* = {*r*_1_, …, *r*_*m*_} with the highest score of $f_{q}^{T}g_{r}$.

### 3.2. Recommendation diversification and determinantal point process

Improving the diversity of recommendations benefits the recommender systems because it introduces novelty and better topic coverage (Ziegler et al., 2005). Many studies on diversification follow the setting of bi-criterion optimization problem, which balances the relevance (between the query and recalled elements) and diversity (Wu et al., 2019). Particularly, diversity can be further divided into two types, (1) individual diversity ^1^ and (2) aggregate diversity ^2^ (Wu et al., 2019). We focus on the individual diversity in this study to adjust the diversity of complementary recommendations, given a user's intent.

The determinantal point process (DPP) is a probabilistic model that is good at modeling repulsion. The recent study (Chen et al., 2018) applies DPP to diversification of item recommendations and develops the fast greedy MAP inference to generate diversified recommendations. Our study is based on DPP with the fast greedy MAP inference in Chen et al. (2018). We introduce details of DPP and the fast greedy MAP inference following the notation in Chen et al. (2018). For the rest of our article, we denote the fast greedy MAP inference as **FG-MAP**.

Formally, the DPP on a discrete set *Z* = {1, 2, …, *M*} is a probability measure P on 2^|*Z*|^ number of subsets of *Z*, where |*Z*| is the number of elements in *Z*. Because the empty set is also a subset of *Z*, when P does not give zero probability to the empty set, there exist a square, positive semidefinite (PSD) and real matrix **L** ∈ ℝ^*M*×*M*^, which satisfies Equation (3) for each subset *Y* ⊆ *Z*.


(3) $$\begin{array}{l} P\left(Y\right)\propto \det\left({\text{L}}_{Y}\right),{\text{L}}_{Y}\in {\mathbb{R}}^{\left|Y|\times |Y\right|} \end{array}$$

**L** serves as a kernel matrix indexed by the elements in *Z* and det(**L**_*Y*_) is the determinant of the sub-matrix extracted from **L** based on elements in *Y*. Equation (3) indicates that the probability of a subset *Y* is proportional to the determinant of the corresponding sub-matrix of the PSD kernel. The MAP inference of the aforementioned DPP P on *Z* is defined in Equation (4).


(4) $$\begin{array}{l} Y_{map}=\arg\underset{Y\subseteq Z}{\max}\det\left({\text{L}}_{Y}\right) \end{array}$$

Unlike the other inference on DPP, the MAP inference of DPP is NP-hard. The algorithm **FG-MAP** approximates the MAP inference in a greedy approach. Equation (5) shows how to greedily select the next candidate item *j* that is added to the existing growing subset *Y*_*g*_ ⊆ *Z* built from the previous iterations. After the current iteration, *Y*_*g*_ grows and *Y*_*g*_: = *Y*_*g*_ ⋃ {*j*} ^3^.


(5) $$\begin{array}{l} j={\arg\max}_{i\in Z\backslash Y_{g}}\log\det\left({\text{L}}_{Y_{g}⋃\left\{i\right\}}\right)-\log\det\left({\text{L}}_{Y_{g}}\right) \end{array}$$

When *Z* becomes the item pools for complementary recommendations *R* = {*r*_1_, …, *r*_*m*_} recalled by the item representations (i.e., item embedding learned by **triple2vec**), the DPP on *R* maximizes the P(Y) and diversifies the recommendations by selecting *r*_*i*_ from *R* iteratively. Now, the kernel matrix **L** could be initialized by the item-to-item similarity matrix based on the item embedding. In our study, we adapt DPP and **FG-MAP**, with **L** defined in Equation (6).


(6) $$\begin{array}{l} \text{L}=\frac{1+H^{T}H}{2},H\equiv \left\{g_{r_{i}}|r_{i}\in R\right\} \end{array}$$

*H* is a sub-matrix of the item embedding for the item pools *R* recalled by the **triple2vec** model. *g*_*r*_*i*__ is normalized embedding of item *r*_*i*_ and the value of *H*^*T*^*H* is shifted to ensure **L** is PSD. We only use one set of items embedding from the **triple2vec** model to compute item similarity, as the distance between *f*_*q*_ and *g*_*r*_ from two sets of embedding represents the complementarity of (*q, r*).

## 4. Diversification strategies

As aforementioned, the diversification strategy for complementary item recommendations in online grocery can fall into two types, heterogenization and homogenization, for exploratory and conventional complementary shopping intent, respectively. In this section, we first introduce the proposed diversification strategies based on DPP. Later, we present our user shopping intent modeling and the selection of diversification strategies with personalization.

### 4.1. Strategy 1: Heterogenization

The heterogenization strategy for complementary item recommendation can be achieved by increasing the diversity in the complementary recommendations *R* recalled by a complementary item recommendation model, i.e., **triple2vec**. It could fulfill the users' intent on exploratory shopping by showing more diverse recommendations. We first generate *R* to ensure complementary recommendations and then re-rank items in *R* to surface more diverse but relevant items to the top. If we do not conduct diversification within the pool of pre-selected complementary items, the diversification logic could easily bias irrelevant items. We can re-rank the items in *R* by modifying **FG-MAP** into bi-criterion optimization. Specifically, we consider the score $S_{q,r_{i}}=\frac{1+f_{q}^{T}g_{r_{i}}}{2}$ for complementarity of (*q, r*_*i*_), where *f*_*q*_ and *g*_*r*_*i*__ are normalized item embeddings. Equation (7) shows the modified objective function for diversification re-rank.


(7) $$\begin{array}{l} r_{j}={\arg\max}_{r_{i}\in R\backslash R_{d}}\underset{\text{complementarity}}{\underset{︸}{\alpha S_{q,r_{i}}}}\\ \;+\underset{\text{increment of diversification}}{\underset{︸}{\left(1-\alpha \right)\left(\log\det\left({\text{L}}_{R_{d}+\left[r_{i}\right]}\right)-\log\det\left({\text{L}}_{R_{d}}\right)\right)}} \end{array}$$

At *t*th iteration, *R*_*t,d*_: = *R*_*t*−1, *d*_ + [*r*_*j*_], where *R*_0,*d*_ = [] and *R*_*t* − 1, *d*_ + [*r*_*j*_] means the newly selected recommendation *r*_*j*_ by the diversification re-rank is inserted at the end of the current item list *R*_*t*−1, *d*_. The weight α controls the amount of diversity introduced to the re-ranked item list. Each selected item *r*_*j*_ can maximize the combined score of diversity and complementarity. The re-ranked item list *R*_*d*_ will surface more diversified recommendation to the top compared with the original item list *R*, in which items are simply sorted by the score *S*_*q*,_*r*__*i*__ in descending order.

### 4.2. Strategy 2: Homogenization

The homogenization strategy is different from the heterogenization strategy. We need to surface more items that are related to the query items but under the same topic, instead of diverse results. For example, assume a query item milk has a list of recommendations *R* = {eggs, cheese, bread, margarine, banana, sausage, yogurt, cereal}. If we want to address the homogenization strategy, the re-ranked recommendations could be *R*_*s*_ = {eggs, cheese, margarine, yogurt, banana, bread, sausage, cereal} ^4^. We encourage more homogeneousness in this strategy while keeping the complementary relationship between recommendations and the query item. *R*_*s*_ surfaces more items under the Dairy & Eggs domain such as cheese and yogurt. The homogenization strategy can be promoted by the similarity of items among the recall set of complementary recommendations. Unlike diversification for the heterogenization strategy which is diverging the item relationship, boosting the homogeneity in the recommendations is more stable. We can mine candidate items in a bigger recall set. Formally, we recall extra complementary items *R*_*x*_ = {*r*_*m*+1_, …, *r*_*n*_} and insert them at the end of *R*. The new item list becomes *R* + *R*_*x*_. To force the similarity between recommendations, we modify the kernel **L** in DPP by Equation (8) and apply DPP to the new dissimilarity matrix **L′**,


(8) $$L^{\prime }=1+\text{diag}(L)-L$$

Where diag(**L**) is a diagonal matrix with all entries in the main diagonal equal to the diagonal of **L** and **1** is a square matrix with all entries equal to 1. Plug **L′** into Equation (7), and we can have a new re-ranking logic on the extended item pool *R* + *R*_*x*_, shown in Equation (9).


(9) $$\begin{array}{l} r_{h}=\arg\max_{r_{i}\in (R+R_{x})\setminus R_{s}}\underset{\text{complementarity}}{\underset{︸}{\beta S_{q,r_{i}}}}\\ \;+\underset{\text{increment of similarity between recommendations}}{\underset{︸}{(1-\beta )\left(\log\det({L^{\prime }}_{R_{s}+[r_{i}]})-\log\det({L^{\prime }}_{R_{s}})\right)}} \end{array}$$

Here, the parameter β is used to control the degree of similarity between recommendations. At *t*th iteration of Equation (9), *R*_*t,s*_: = *R*_*t* − 1, *s*_ + [*r*_*h*_], where *R*_0, *s*_ = []. Both Equations (7), (9) can be optimized by the **FG-MAP** algorithm mentioned in Chen et al. (2018)^5^.

### 4.3. User intent modeling and diversification strategy selection

Only having two re-ranking strategies is not enough because we need to figure out the selection of two diversified re-ranking strategies for a certain user. We leverage the heuristic that users who usually add more diverse items during the next-*k* purchases would prefer more exploratory complementary shopping with the heterogenization strategy, while users who commonly add less diverse items during the next-*k* purchases would prefer more conventional complementary shopping with the homogenization strategy.

Formally, given a query item *q* at time *t* and a list of next-*k* items *B*_*q*_ = {*b*_*t*+1_, …, *b*_*t*+*k*_} purchased by a user *u*, we leverage the taxonomy information *tax*(·) ^6^ to estimate how much diversity the user *u* prefers. Let *B*_*T,q*_ = [*tax*(*b*_*t*+1_), …, *tax*(*b*_*t*+*k*_)] be the list of departments of the next-*k* items purchased by the user and |*B*_*T,q*_| be the number of unique elements in *B*_*T,q*_. We can estimate the degree of diversity for the query item *q* and the user *u* in Equation (10).


(10) $$\begin{array}{l} z_{u,q}=\frac{\left|B_{T,q}\right|}{k} \end{array}$$

However, the score *z*_*u,q*_ is at user-item level and not stable due to the sparsity issue. We then extend it to a score at user-department level to reduce the sparsity, as shown in Equation (11), where *dept*_*i*_ is the department *i* and the score *z*_*u, dep*_*t*__*i*__ is an average of score *z*_*u,q*_ for any query items satisfying *tax*(*q*) = *dept*_*i*_.


(11) $$\begin{array}{l} z_{u,dept_{i}}=\frac{1}{N}\overset{N}{\underset{\left\{q|tax\left(q\right)=dept_{i}\right\}}{\sum }}z_{u,q} \end{array}$$

We treat the score *z*_*u, dep*_*t*__*i*__ as the user intent score of exploratory complementary shopping and apply a threshold *T* ∈ [0, 1] to binarize *z*_*u, dep*_*t*__*i*__ learnt from the training data. If *z*_*u, dep*_*t*__*i*__ < *T*, the user *u* prefers the more conventional complementary shopping under the department *dept*_*i*_, otherwise, the user *u* might prefer more exploratory complementary items because *u* tends to add items from different departments during the next-*k* purchases. We can combine the score *z*_*u, dep*_*t*__*i*__ with the heterogenization and homogenization strategies to develop a dynamic re-ranking algorithm for complementary item recommendations (summarized in Algorithm 1), It provides either diversified re-ranking strategy based on the user intent on a certain department *dept*_*i*_ of the query item *q*.

**Algorithm 1:** Dynamic re-ranking of complementary recommendation with user intent.

**Require:** *u*, *q*, *R*, *R*_*x*_, α, β, *T*, *z*_0_, *k*;	
**Ensure:**	
1:	*R*_*out*_ = []
2:	*dept*_*i*_ = *tax*(*q*)
3:	**if** *z*_*u, dep*_*t*__*i*__ available **then**
4:	use *z*_*u, dep*_*t*__*i*__
5:	**else**
6:	*z*_*u, dep*_*t*__*i*__ = *z*_0_
7:	**end if**
8:	**if** *z*_*u,dep*_*t*__*i*__ > *T* **then**
9:	use *R*, α to compute *R*_*d*_ by Equation (7) and **FG-MAP** with *k* iterations (heterogenization strategy)
10:	*R*_*out*_: = *R*_*d*_
11:	**else**
12:	use *R* + *R*_*x*_, β compute *R*_*s*_ by Equation (9) and **FG-MAP** with *k* iterations (homogenization strategy)
13:	*R*_*out*_: = *R*_*s*_
14:	**end if**
15:	**return** *R*_*out*_ as the re-ranked complementary recommendations for *u* and *q*

We add *z*_0_ as a default value for cold departments of query items which are not seen in the history. *z*_0_ could be initialized by the average of all *z*_*u, dep*_*t*__*i*__.

## 5. Evaluation

In this section, we evaluate our proposed solution on the publicly available Instacart dataset (Instacart, 2017). We also conduct a parameter analysis of re-ranking performance with different *T*.

### 5.1. Evaluation setting

The Instacart dataset (Instacart, 2017) has 49,677 distinct items, 134 distinct aisles, 21 distinct departments, and 206,209 distinct users. We train the **triple2vec** model on the Instacart training dataset, with an embedding dimension of 100, a batch size of 128, an initial learning rate of 0.05, and a stochastic gradient descent optimizer. We also compute *z*_*u, dep*_*t*__*i*__ for each pair of (*u, dept*_*i*_) for the next-5 purchase (*k* = 5 in Equation 10). When evaluating the re-ranking strategies, we compare the results before and after the re-rank. Given a query item *q*, a user *u*, and recommendations *R, R*_*x*_ generated by the **triple2vec** model, we compute the Hit-Rate@5 and Normalized Discounted Cumulative Gain (NDCG@5) for raw complementary recommendation *R* and the re-ranked complementary recommendations by (1) heterogenization only, (2) homogenization only, and (3) combining heterogenization and homogenization strategies with user intent scores dynamically on the task of next-item prediction. The reason why we focus on the next-5 purchase is because the user intent might last for a short period and we want to study the impact of two different complementary recommendations on the top recommendations. If we consider bigger *k*, it is likely to introduce diversity in recommendations. Here, we define *R* = {*r*_1_, *r*_2_, *r*_3_, *r*_4_, *r*_5_} and *R*_*x*_ = {*r*_6_, *r*_7_, *r*_8_, *r*_9_, *t*_10_} to cooperate the metrics of Hit-Rate@5 and NDCG@5.

Since it is a novel study on exploratory vs. non-exploratory user behaviors for complementary item recommendations, it is hard to find proper baselines. We choose three baseline models for comparison. (1) As aforementioned, we only consider the transaction data due to its high accessibility, we consider the raw recommendations from ***triple2vec*** for pure complementary item recommendations. (2) The second baseline model is the diversified recommendation by DPP for the pure heterogenization strategy. (3) Similarly, we use *T* = 1 to force homogenization and generate our third baseline model for comparisons.

To further understand the trade-off between heterogenization and homogenization strategies, we evaluate the combined strategy with *T* ∈ {0.1, 0.2, 0.3, 0.4, 0.5, 0.6, 0.7, 0.8, 0.9}. We use α = β = 0.01 for evaluations.

### 5.2. Evaluation results

We evaluate our re-ranking strategies on the Instacart evaluation dataset and the detailed results are shown in Table 1. The heterogenization strategy improves the Hit-Rate@5 and NDCG@5 compared with the raw recommendations, while only using homogenization strategy reduces the performance. Combining both re-ranking strategies together with a proper *T* improves the overall performance. Particularly, *T* = 0.2 achieves the best Hit-Rate@5 with and *T* = 0.1 achieves the best NDCG@5. This result is reasonable because *T* = 0.2 means, on average, users purchase the next-5 items under the same department. The evaluation results show a better performance for covering users who prefer conventional complementary shopping with the homogenization strategy.

**Table 1:** Detailed results of next-item prediction.

	**Hit-Rate@5**	**NDCG@5**
Raw recommendation (triple2vec)	0.05581	0.03216
*T* = 0 (heterogenization only by DPP)	0.05581	0.03379
*T* = 0.1	0.05612	0.03380
*T* = 0.2	0.05625	0.03377
*T* = 0.3	0.05612	0.03371
*T* = 0.4	0.05558	0.03318
*T* = 0.5	0.05388	0.03214
*T* = 0.6	0.05259	0.03133
*T* = 0.7	0.05261	0.03128
*T* = 0.8	0.05261	0.03127
*T* = 0.9	0.05261	0.03127
*T* = 1 (homogenization only)	0.05261	0.03127

Hit-Rate@5 and NDCG@5 are shown. The highest score is highlighted by underline.

Note that, only applying the homogenization strategy reduces both Hit-Rate@5 and NDCG@5. It might be because only showing complementary recommendations in a narrow scope is likely to miss users' interests (see Section 5.3 for more details). If a user is not interested in the first recommended item, this user will likely not be interested in the following recommendations because they are similar. The heterogenization strategy improves this feature by surfacing different complementary items to the top. Now, the re-ranked recommendations are more likely to hit this user's interests. Combining these two strategies together actually covers the requirements of exploratory and conventional complementary shopping intents from users.

In summary, our results show that combining two strategies dynamically improves the overall performance, compared with only using a single diversification strategy.

### 5.3. User intent modeling

**Figure 3:**
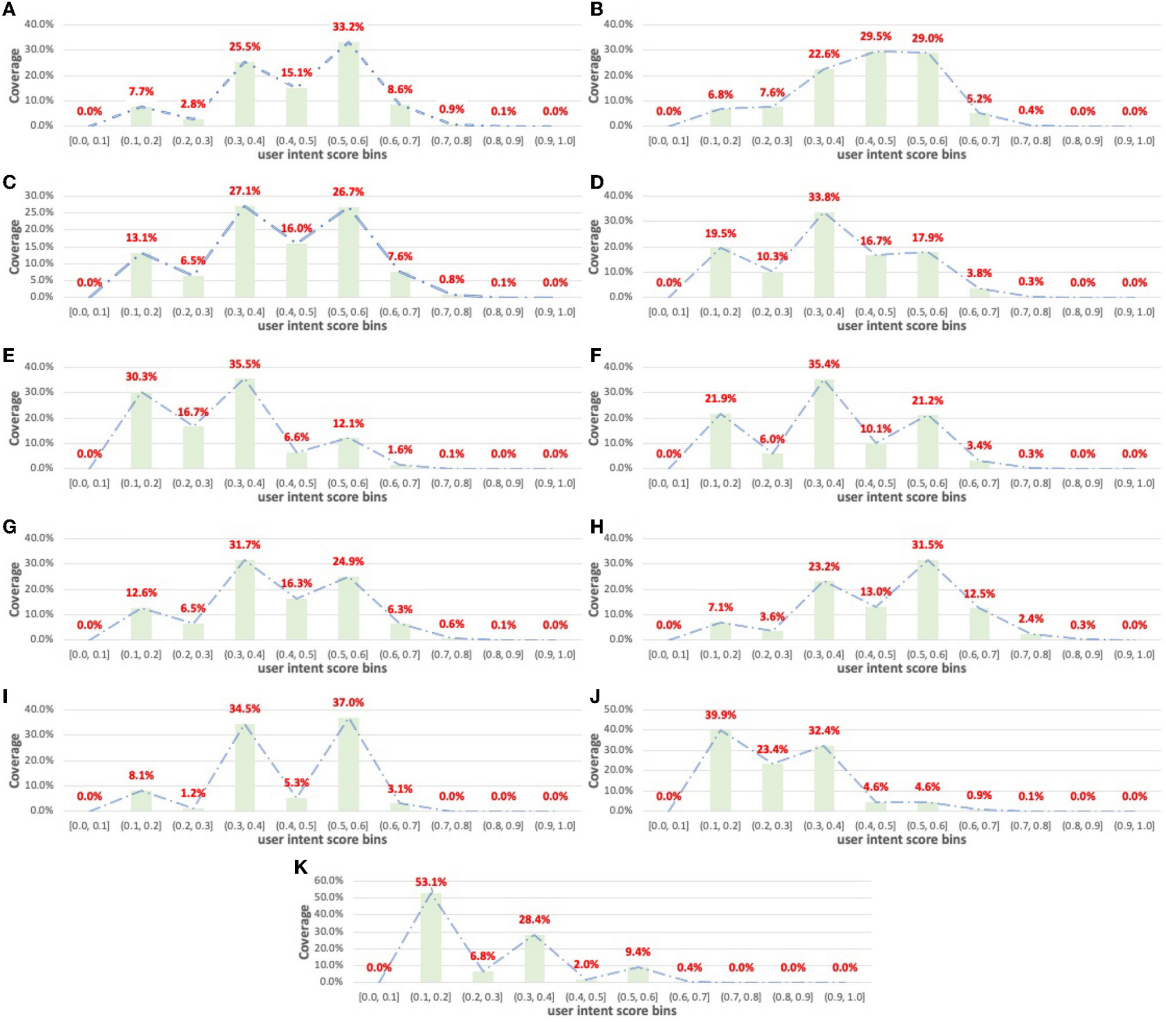
Distribution of user intent scores by departments. **(A)** Deli, **(B)** Produce, **(C)** Dair eggs, **(D)** Beverage, **(E)** Meat seafood, **(F)** Canned goods, **(G)** Frozen, **(H)** Bakery, **(I)** Breakfast, **(J)** Pantry, and **(K)** Household.

To further indicate the necessity of personalized diversification strategies for complementary item recommendations, we visualize the distribution of user intent score *z*_*u, dep*_*t*__*i*__ by departments. Figure 3 summarizes the distribution. We can see that for a given department, the user intent scores distribute differently. For example, the majority of the user intent scores in Deli and Produce departments follow in the range [0.3, 0.6], which indicates that users tend to shop more diverse items when the query items are from these departments. Dairy and Beverage have similar results such as Deli and Produce. However, for departments such as Household and Pantry, the majority of the user intent scores are in the range [0.1, 0.4]. Compared with other departments, the users tend to purchase more homogeneous items when the query items are from Household and Pantry, which is reasonable because these departments usually cover most of the department-related shopping demands and correlate less with other departments. The aforementioned observation addresses the requirement of tuning the diversification of complementary item recommendations with personalization.

## 6. Conclusion and future work

We focus on the re-ranking of complementary recommendations in online grocery and point out the exploratory and conventional complementary shopping intents from users. To fulfill these two user intents, we propose two re-ranking strategies, heterogenization and homogenization, based on DPP on the raw complementary recommendations and dynamically combine two re-rankings as a final solution to improve the performance. We demonstrate the effectiveness of our solution on the publicly available Instacart dataset.

## Data availability statement

The original contributions presented in the study are included in the article/supplementary material, further inquiries can be directed to the corresponding author.

## Author contributions

NS helped the experiments. JC, SK, and KA help the iteration of the research ideas and the design during this research. All authors contributed to the article and approved the submitted version.

## Footnotes

^1^Individual diversity refers to the diversity of recommendations for a given user or individual diversity focuses on the problem of how to maximize item novelty in the face of already recommended ones when generating the recommendation list.

^2^Aggregate diversity refers to the diversity of recommendations across all users or aggregate diversity can be viewed as a problem of how to improve the ability of a recommender system to recommend long-tail items.

^3^For more details of the fast greedy MAP inference algorithm, please refer to Chen et al. (2018).

^4^However, the diversification re-rank aforementioned could result in *R*_*d*_ = {eggs, banana, cheese, bread, sausage, cereal, margarine, yogurt}.

^5^Algorithm 1 in Chen et al. (2018).

^6^*tax*(·) returns the department of the input item.
